# Central Composite Optimization of Glycerosomes for the Enhanced Oral Bioavailability and Brain Delivery of Quetiapine Fumarate

**DOI:** 10.3390/ph15080940

**Published:** 2022-07-29

**Authors:** Randa Mohammed Zaki, Munerah M. Alfadhel, Manal A. Alossaimi, Lara Ayman Elsawaf, Vidya Devanathadesikan Seshadri, Alanood S. Almurshedi, Rehab Mohammad Yusif, Mayada Said

**Affiliations:** 1 Department of Pharmaceutics, College of Pharmacy, Prince Sattam Bin Abdulaziz University, P.O. Box 173, Al-Kharj 11942, Saudi Arabia; m.alfadhel@psau.edu.sa; 2 Department of Pharmaceutics and Industrial Pharmacy, Faculty of Pharmacy, Beni-Suef University, Beni-Suef P.O. Box 62514, Egypt; 3 Department of Pharmaceutical Chemistry, College of Pharmacy, Prince Sattam Bin Abdulaziz University, P.O. Box 173, Al-Kharj 11942, Saudi Arabia; m.alossaimi@psau.edu.sa (M.A.A.); lolahappy71@yahoo.com (L.A.E.); 4 Department of Pharmacology and Toxicology, College of Pharmacy, Prince Sattam Bin Abdulaziz University, P.O. Box 173, Al-Kharj 11942, Saudi Arabia; v.adri@psau.edu.sa; 5 Department of Pharmaceutics, College of Pharmacy, King Saud University, P.O. Box 2457, Riyadh 11451, Saudi Arabia; marshady@ksu.edu.sa; 6 Department of Pharmaceutics, Faculty of Pharmacy, Mansoura University, Mansoura P.O. Box 35516, Egypt; rehabyusif@yahoo.com; 7 Department of Pharmaceutics and Pharmaceutical Technology, College of Pharmacy, Taibah University, P.O. Box 30039, Al-Madinah Al-Munawarah 41477, Saudi Arabia; 8 Department of Pharmaceutics and Industrial Pharmacy, Faculty of Pharmacy, Cairo University, Cairo P.O. Box 11562, Egypt; mayada.mohamed@pharma.cu.edu.sa

**Keywords:** schizophrenia, quetiapine fumarate, glycerosomes, central composite rotatable design, bioavailability, pharmacokinetic

## Abstract

This study aimed to formulate and statistically optimize glycerosomal formulations of Quetiapine fumarate (QTF) to increase its oral bioavailability and enhance its brain delivery. The study was designed using a Central composite rotatable design using Design-Expert^®^ software. The independent variables in the study were glycerol % *w*/*v* and cholesterol % *w*/*v*, while the dependent variables were vesicle size (VS), zeta potential (ZP), and entrapment efficiency percent (EE%). The numerical optimization process resulted in an optimum formula composed of 29.645 (*w*/*v*%) glycerol, 0.8 (*w*/*v*%) cholesterol, and 5 (*w*/*v*%) lecithin. It showed a vesicle size of 290.4 nm, zeta potential of −34.58, and entrapment efficiency of 80.85%. The optimum formula was further characterized for DSC, XRD, TEM, in-vitro release, the effect of aging, and pharmacokinetic study. DSC thermogram confirmed the compatibility of the drug with the ingredients. XRD revealed the encapsulation of the drug in the glycerosomal nanovesicles. TEM image revealed spherical vesicles with no aggregates. Additionally, it showed enhanced drug release when compared to a drug suspension and also exhibited good stability for one month. Moreover, it showed higher brain C_max_, AUC_0–24_, and AUC_0–∞_ and plasma AUC_0–24_ and AUC_0–∞_ in comparison to drug suspension. It showed brain and plasma bioavailability enhancement of 153.15 and 179.85%, respectively, compared to the drug suspension. So, the optimum glycerosomal formula may be regarded as a promising carrier to enhance the oral bioavailability and brain delivery of Quetiapine fumarate.

## 1. Introduction

Schizophrenia is a chronic and severe mental disorder, defined by positive (hallucinations and delusions), negative (disruption of normal behavior and emotion), and cognitive (difficulties in memory and attention) symptoms [1]. Symptoms of schizophrenia start in adulthood and continue throughout life [2]. These symptoms can be managed by an antipsychotic drug, especially atypical antipsychotic drugs [2]. Among atypical antipsychotic drugs Quetiapine fumarate (QTF), QTF, is a second-generation atypical antipsychotic drug that has broader efficiency than traditional antipsychotics and many other atypical antipsychotic drugs [3]. It is a dibenzothiazepine derivative [2]. It is effective against positive and negative symptoms of schizophrenia with good neurocognition properties [4,5]. The exact mechanism of action of QTF is unknown, but it is thought to block nerve receptors for many neurotransmitters, restricting communications between nerves. This action could be done by combining dopamine type 2 and serotonin type 2 (5HT2) receptor antagonism. QTF also has an antidepressant activity which could be due to the effect of its metabolite N-desalkyl quetiapine fumarate on selective norepinephrine reuptake inhibition and 5-HT1A and 5-HT7 receptor activity [6]. Many clinical trials showed that QTF has an acceptable safety profile [7]. It was approved for first-line treatment of schizophrenia [8]. It also showed effectiveness in bipolar mania [9].

The oral route is the most common route for drug delivery, but many factors may affect drug absorption and bioavailability, like pH of the GIT, drug solubility, residence time, and hepatic first-pass metabolism [10]. QTF has an oral bioavailability of 9%, which is related to its high hepatic metabolism resulting in reduced brain concentration [11]. QTF suffers from low water solubility, especially at higher pH, and as a result, reduced absorption is anticipated at higher pH [12]. QTF has a plasma half-life of 6 h, and as a result, it needs frequent dosing to maintain effective therapeutic concentration [12]. Schizophrenia treatment via the oral route is very challenging due to the presence of a protective blood-brain barrier (BBB), complex tight junctions that make sealing for the paracellular pathway, and P-glycoprotein, which reduces the amount delivered of many drugs into the brain. QTF is a P-glycoprotein substrate that suffers from reduced brain concentration following oral administration [13]. Therefore, the incorporation of QTF in lipid-based nanoformulations like glycerosomes may overcome the overmentioned limitations. GLSMs could protect the encapsulated drug from degradation in the GIT [14]. They also can target the lymphatic system owing to their lipid nature [15,16]. Many drugs could be orally delivered through the lymphatic system, which avoids hepatic first-pass metabolism [17,18]. It was reported that lipid-containing nanoparticles could enhance the uptake of drugs into the lymphatic circulation, which could be related to their small size and lipid nature [14].

Nanoformulations have many advantages for brain delivery like their flexibility [19], increased solubility of drugs [20], the release of the drug in a controlled manner [21], crossing and overcoming the BBB, and targeting the drugs into the brain [19], which is desired for drugs treating mental illness like schizophrenia [22]. This results in increasing the concentrations of drugs in the brain tissues and cells with the consequence of increasing their bioavailability [22].

Glycerosomes (GLSMs) are a new generation of liposomes containing phospholipids, water, and varying concentrations of glycerol (10–30 *w*/*v*%) [23]. Glycerol is non-toxic, harmless, and non-irritating and so is safely used. GLSMs can encapsulate both hydrophilic and hydrophobic drugs [24]. They differ from liposomes by being more stable and having greater fluidity than liposomes [23]. The increased fluidity of GLSMs is related to the presence of glycerol in high percent, which makes modifications to the bilayer membrane [25]. This increased fluidity can aid in better penetration into the brain tissue. GLSMs may contain cholesterol which increases the stability of the bilayer [25]. GLSMs are prepared by the same common techniques used for liposome preparations [23,26].

Our work aimed to develop QTF glycerosomes to enhance the oral bioavailability and brain delivery of QTF.

## 2. Results and Discussion

### 2.1. Evaluation of QTF Glycerosomal Formulations

#### 2.1.1. Measurement of Vesicle Size VS, PDI, and ZP

**Figure 1:**
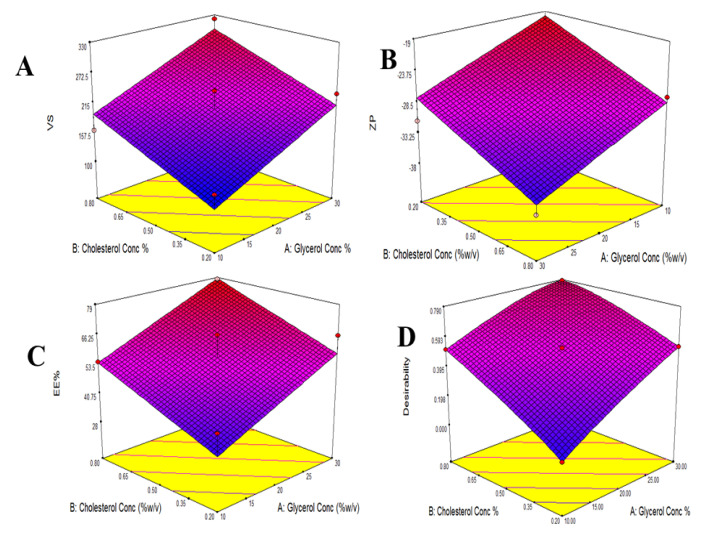
Response surface plot for the effect of Glycerol concentration and Cholesterol concentration on VS (**A**), ZP (**B**), EE% (**C**), and Desirability (**D**).

**Figure 2:**
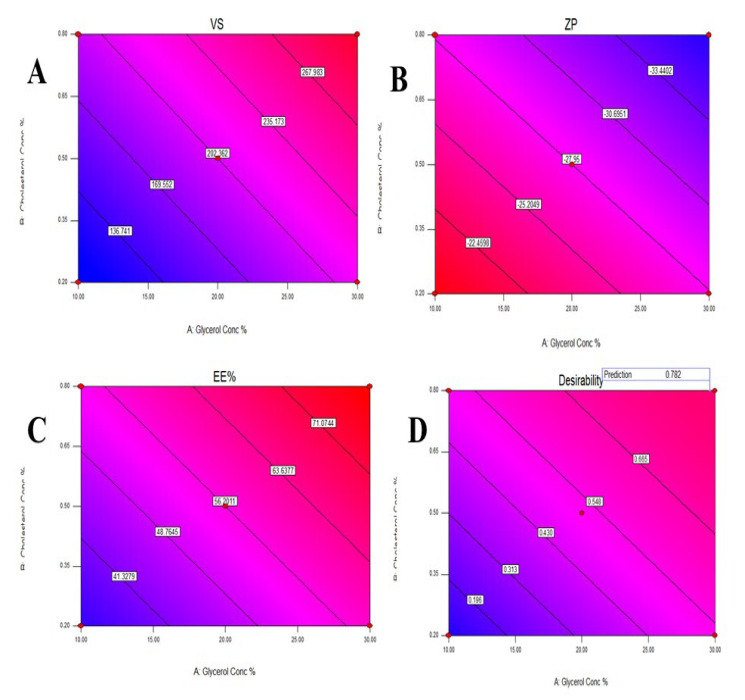
Contour plot for the effect of Glycerol concentration and Cholesterol concentration on Vesicles size (**A**), Zeta potential (**B**), Entrapment efficiency% (**C**), and Desirability (**D**).

The VS of various glycerosomal formulations varied between 110.23 to 321.51 nm, as evident in Table 1. The smaller the particle, the larger the surface area available for drug absorption and penetration into the brain [27]. The effects of Glycerol concentration (X1) and cholesterol concentration (X2) on VS are shown in Figure 1A and Figure 2A.

The linear model was the most suitable one to be fitted to VS data (*p*-value = 0.0041) with a small difference between the adjusted and predicted R^2^ (less than 0.2), which ensures the validity of the model [28] and high adequate precision of 9.66 (greater than 4); this indicated that the model was able to navigate the design space as shown in Table 2 [29].

The effect of the studied factors on VS could be studied using the following equation:

VS = 202.36 + 53.64 X1 + 44.79 X2(1)

It was evident from ANOVA analysis, as represented in Table 3, that both Glycerol concentration (X1) and cholesterol concentration (X2) significantly affected VS values with (*p*-values = 0.0051 and 0.0115, respectively), where the increase in both glycerol and cholesterol concentrations led to a significant increase in VS as revealed by the positive sign of their coefficients in the correlation equation. However, as per Equation (1), the high regression coefficient of Glycerol concentration (X1) indicated a higher impact than cholesterol concentration (X2) on VS.

The increase in VS with the increase in glycerol concentration could be explained by the sticky nature of glycerol. It increased the viscosity of the dispersion, which made it difficult for size reduction during sonication. Moreover, it loosens the packing of the glycerosomal lipid bilayer membrane, which results in decreased curvature of the bilayer, and as a result, bigger vesicles are formed [23,26].

Moreover, it was noted that at lower concentrations of cholesterol, the order of the lipid bilayer chain is increased, which resulted in close packing and, as a result, the size decreased. However, due to its hydrophobic nature, increasing its concentration led to increased hydrophobicity of the bilayer and disturbance of the lipid membrane of GLSMs and, as a result, increased vesicle size in an attempt to reach thermodynamic stability. In addition, cholesterol increased the rigidity of the GLSMs membrane, which resulted in resistance to size reduction during the sonication step [30]. This explained the positive impact of cholesterol on vesicle size.

PDI points out the magnitude of the size diversity and is expressed by values between 0 and 1 [29]. As shown in Table 1, the PDI values of the prepared glycerosomal formulations lay between 0.13 and 0.40; this could indicate that the size distribution was within the acceptable limits for the prepared glycerosomal dispersions [29].

ZP indicates the physical stability of the glycerosomal formulations. The larger the value of the ZP, The larger the repulsion forces between vesicles, which resulted in reduced aggregation and increased stability of the system [31].

As shown in Table 1, The ZP of different glycerosomal formulations ranged from −19.1 to −37.7 mV. This could point out that the prepared glycerosomal formulations were physically stable [29]. The effects of Glycerol concentration (X1) and cholesterol concentration (X2) on ZP are illustrated in Figure 1B and Figure 2B.

The data of ZP were fitted to a linear model (*p*-values < 0.0058) with an adequate high precision (9.1957) and a difference between the adjusted and predicted R^2^ of less than 0.2. The following equation could relate the effect of the studied factors on ZP:

ZP = 27.95 + 4.05 X1 + 4.19 X2(2)

It was shown from the ANOVA analysis in Table 3 that both glycerol (X1) and cholesterol (X2) concentrations significantly affected ZP (*p*-values = 0.0109 and 0.0094, respectively). Both X1 and X2 significantly increased the ZP absolute values and this was confirmed by their positive regression coefficients as per Equation (2). However, cholesterol concentration (X2) showed a higher impact on ZP values than glycerol concentration (X1) due to its higher regression coefficient value as in Equation (2).

Increasing ZP absolute values with increasing glycerol concentration could be related to its interaction with polar heads of the phospholipids in the lipid bilayer which resulted in a change in the orientation of molecules and affected the total surface charge of the vesicles [32]. Furthermore, The rise in the ZP absolute values with the increase in cholesterol concentration could be due to its ability to modify the surface charge of the vesicles preventing vesicle aggregation and increasing their stability [24,33].

#### 2.1.2. Measurement of EE%

The EE% of various glycerosomal formulations varied between 28.3 to 78.1%, as shown in Table 1, confirming successful encapsulation for the drug in the GLSMs so that glycerol-containing nanovesicles can be utilized as a successful delivery system for QTF. The effects of glycerol concentration (X1) and cholesterol concentration (X2) on EE% are represented in Figure 1C and Figure 2C.

The EE% data were best analyzed using a linear model (*p*-values < 0.0095) where the adequate precision is high (8.1482). In addition, a less than 0.2 difference between the adjusted and predicted R^2^ was found. The following equation could make a relationship between the studied factors on EE%:

EE% = 56.2 + 12.14 X1 + 10.17 X2(3)

It was evident from the ANOVA analysis that both glycerol and cholesterol concentrations significantly affected the EE% (*p*-values = 0.0111 and 0.0230, respectively), where both had positive impacts on the EE% values. However, Glycerol concentration (X1) showed a higher impact than cholesterol concentration (X2) on EE% due to its higher regression coefficient as per Equation (3).

The increase in EE% with the increase in Cholesterol concentration could be referred to as the lipophilic nature of the drug, which increased its integration in the lipid phase containing lipophilic cholesterol [26]. Additionally, Cholesterol increases the rigidity of the lipid bilayer membrane, controls permeability, and enhances vesicle stability [26]. So, increasing cholesterol concentrations reduced the leakage of the entrapped drug, consequently enhancing the EE%.

Output data of Central Composite Design of QTF loaded GLSMs is shown in Table 2. ANOVA for Central Composite Design of QTF loaded GLSMs is shown in Table 3.

### 2.2. Statistical Analysis, Optimization, and Validation

**Figure 3:**
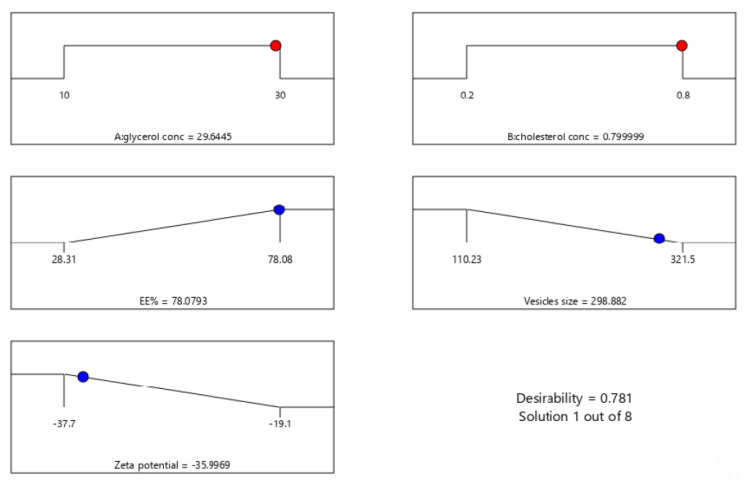
The composition of the optimized formula and its predicted responses according to Central Composite Design.

Design Expert^®^ software was used to perform A numeric analysis to make a selection of an optimum glycerosomal formulation, where VS was minimized while ZP and EE% were maximized. This optimization process showed an optimum glycerosomal formulation with a desirability of 0.781 (Figure 1D and Figure 2D). It was composed of 29.645 (*w*/*v*%) glycerol, 0.799 (*w*/*v*%) cholesterol and 5 (*w*/*v*%) lecithin. The predicted values of VS, ZP, and EE% were 298.882 nm, −35.997 mV, and 78.079%, respectively, as shown in Figure 3. The optimum formula was then prepared, followed by its validation as demonstrated in Table 4 with a % relative error of less than 5% from the predicted values shown by the design expert software, indicating the fitness of the model [34].

### 2.3. Evaluation of the Optimum QTP Formula

#### 2.3.1. Differential Scanning Calorimetry (DSC)

**Figure 4:**
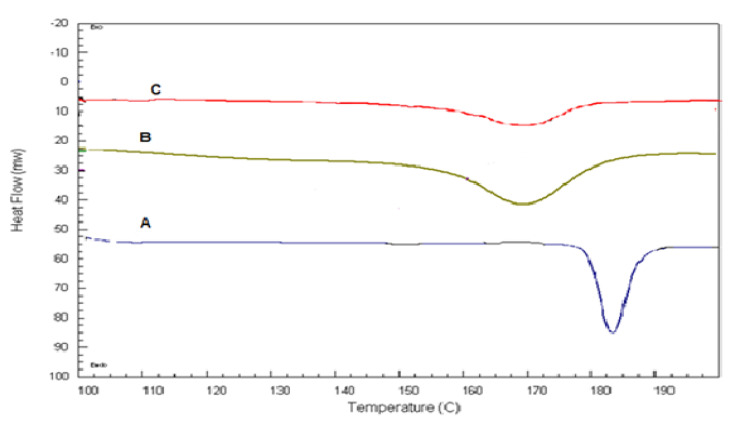
DSC thermograms of A: Pure QTF, B: lecithin, cholesterol, and QTF physical mixture, C: the optimized formula.

DSC thermograms of pure QTF, a physical mixture of lecithin, cholesterol, and QTF, and the optimum glycerosomal formula are shown in Figure 4. Pure QTP showed a sharp endothermic peak at 182.95 °C, indicating its melting point in crystal form (Figure 4A) [35]. The drug’s endothermic peak was well preserved in its physical mixture with lecithin and cholesterol (Figure 4B) with changes in the form of shifting of the temperature of the melt or broadening. It is familiar that the quantity of materials used, particularly in drug excipient mixtures, may affect the peak shape and enthalpy. So, these small changes in the melting endotherm of the drug may be resulted from mixing the drug with the excipients, which reduced the purity of each component in the mixture and this may not necessarily refer to potential incompatibility [10,36,37]. Therefore, it could be concluded that QTF is compatible with excipients used in the formulation. In addition, the optimum glycerosomal formula (Figure 4C) showed a broad endothermic peak with a decrease in the intensity, indicating encapsulation for the drug and its conversion into an amorphous form. Besides, changes in the drug crystallinity may lead to shifts in the melting point [38].

#### 2.3.2. X-ray Diffraction Study (XRD)

**Figure 5:**
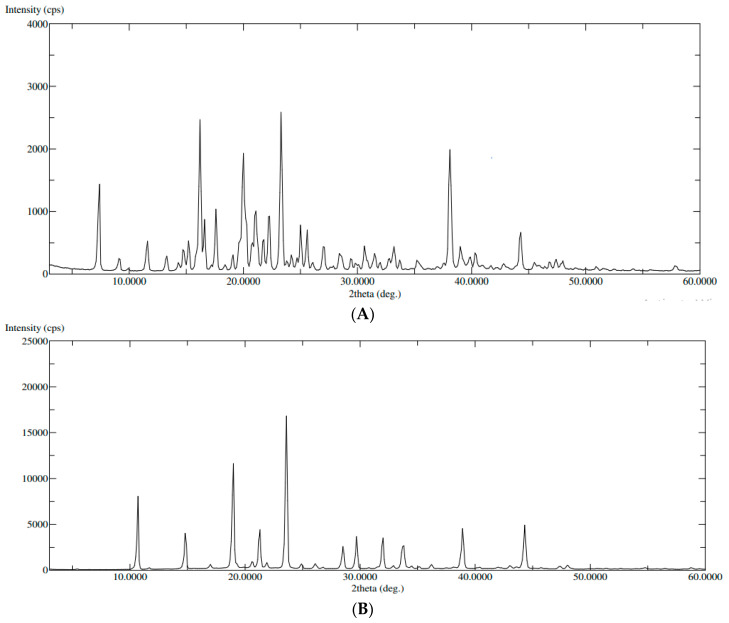
XRD of (**A**): pure QTF, (**B**): the optimized formula.

XRD spectra of pure QTF and the optimum formula were illustrated in (Figure 5). The XRD of pure QTF showed sharp peaks at 20 Ө scattered angles of 16°, 20°, 21°, 22°, and 23° indicating its crystalline nature (Figure 5A). However, a decrease in the intensity of some drug peaks and disappearance of others was noted in the XRD spectrum of the optimized formula (Figure 5B), probably due to the encapsulation of the drug within GLSMs nanovesicles. These results support the prediscussed DSC results [39].

#### 2.3.3. Transmission Electron Microscopy (TEM)

**Figure 6:**
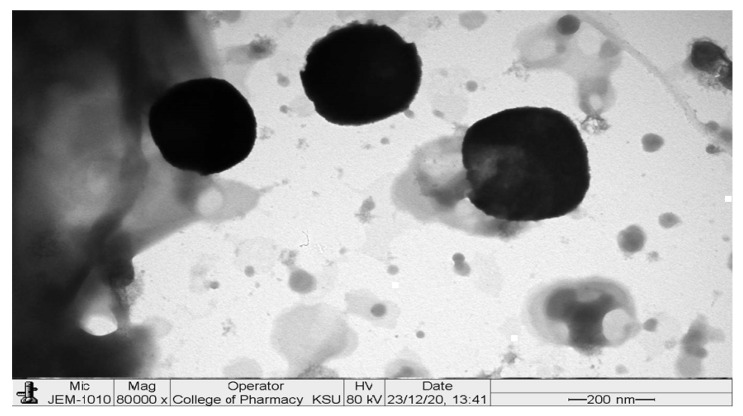
TEM image of the optimized formula.

TEM image showed small spherical vesicles, as shown in Figure 6. There were no aggregates that indicated good physical stability of the dispersion and could be related to the high ZP on the surfaces of the vesicles, which induces repulsion between the adjacent GLSMs [29,40,41,42]. Moreover, GLSMs showed an average dimension of 272.83 ± 36.21 nm.

#### 2.3.4. In-Vitro Release

**Figure 7:**
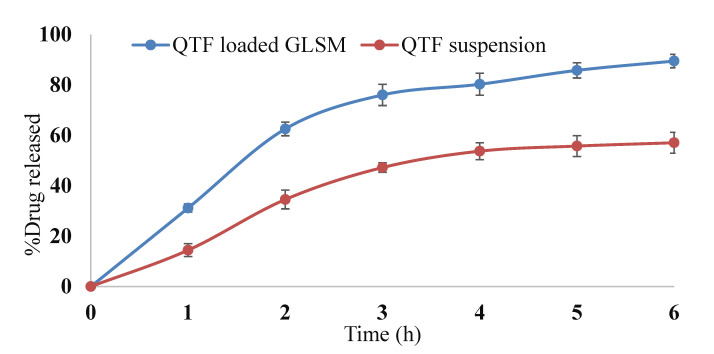
In vitro release profile of QTF from QTF loaded GLSMs and QTF suspension.

Figure 7 shows the release profile of the optimum GLSM formula in comparison to QTF suspension. The optimum formula showed enhanced QTF release in comparison to the drug suspension. This could be related to the amphiphilic properties of phosphatidylcholine used in glycersomes formation [26,43]. Moreover, the reduction in vesicle size of the glycerosomal formulation may increase the drug release. Vesicle size affects drug release from GLSMs, where a higher release rate was obtained by smaller vesicles than larger sized ones [26,44]. Our results comply with the results obtained by Salem et al., who showed a significant enhancement of the release of drugs from GLSMs in comparison to drug suspension [26].

#### 2.3.5. Effect of Aging

Table 5 demonstrates the effect of storage for one month on the stability of the optimum glycerosomal formula. There were no significant changes in VS, ZP, and EE% at all-time points (7 and 30 days), which indicates good stability of the optimum GLSM formula during storage for one month at 4 °C. The slight decrease in EE% may be due to the presence of glycerol which enhances the flexibility and loosen the packing of the glycerosomal lipid bilayer, which results in leakage of drug from GLSMs. However, there was a slight increase in vesicle size, which may be attributed to the hydrophilic nature of glycerol, so an increase in the water uptake of the vesicle bilayers thus increases vesicle size.

### 2.4. In-Vivo Bioavailability of the Optimized QTP Glycerosomal Formula

**Figure 8:**
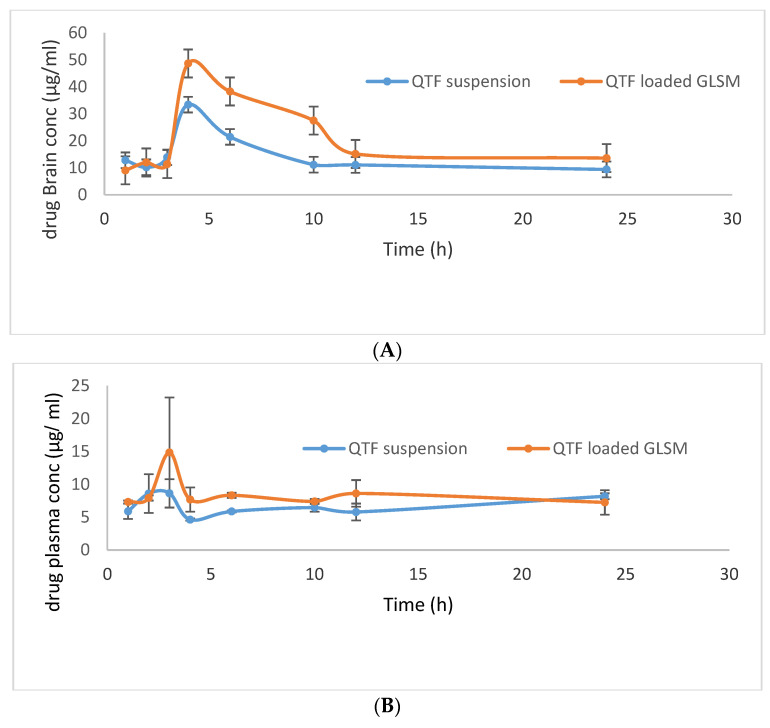
QTF mean brain concentration (**A**) and mean plasma concentration (**B**) after oral administration of QTF suspension, and QTF Loaded GLSMs.

The mean QTF concentrations in rat brains and plasma upon administration of the optimum GLSM formula and aqueous drug suspension are shown in Figure 8. The optimum GLSM formula showed a significantly higher brain C_max_, AUC_0–24_, and AUC_0–∞_ in comparison to QTF suspension with *p*-values of 0.0477, 0.003, and 0.003, respectively, as pointed out in Table 6. It also showed a significantly higher plasma AUC_0–24_ and AUC_0–∞_ in comparison to QTF suspension with *p*-values of 0.004 and 0.049, respectively, as shown in Table 6. The optimum GLSM formula showed brain and plasma bioavailability enhancement of 153.15 and 179.85%, respectively, compared to the drug suspension [29]. These obtained findings could indicate the ability of the optimum GLSM formula to enhance the oral bioavailability and brain delivery of QTF in comparison to a drug suspension, which could be related to the lipophilic nature of the formula, which reduced first-pass metabolism [45,46]. In addition to the enhanced solubility of QTF within the formula, the small vesicle size of the optimum GLSM formula and increased fluidity of the GLSM lipid bilayer membrane due to the presence of glycerol which led to better penetration into the brain tissue. It was also reported that polar lipids such as phospholipids are associated with proteins in the structural membranes due to the structural similarity with biomembranes, which resulted in facilitating drug transport across BBB [11,47].

## 3. Materials and Methods

### 3.1. Materials

Quetiapine fumarate (QTF) was gifted by the Al jazeera pharmaceuticals Co. Lecithin, cholesterol, and glycerol were purchased from Sigma-Aldrich (Saint Louis, MO, USA). Acetonitrile for HPLC ≥ 99.9% (Sigma-Aldrich^®^, Saint Louis, MO, USA). Methanol HPLC grade, Diethyl ether HPLC grade, and Chloroform HPLC grade were purchased from (Sigma-Aldrich^®^, Saint Louis, MO, USA). HPLC grade water was obtained from a Milli-Q ultrapure Water system. Orthophosphoric acid for HPLC 85–90% (Fluka^®^, Buchs, Switzerland). Sodium hydroxide pellets (Sigma-Aldrich^®^, Saint-Quentin-Fallavier, France). Nylon membrane filter type 0.45 μm HNWP was purchased from (Merck, Darmstadt, Germany).

### 3.2. Statistical Design of QTF Loaded GLSMs

This study was designed using a central composite rotatable design to address the effect of different variables of formulation on QTF-loaded GLSMs characteristics using Design Expert^®^ software (Ver. 7, Stat-Ease, Minneapolis, MN, USA). The independent variables were glycerol concentration (X1) which ranged from 10 to 30 *w*/*v%* and cholesterol concentration (X2), which lay between 0.2 to 0.8 *w*/*v%*. This resulted in 9 experimental runs. QTF was kept constant in all formulations at a concentration of 1 *w*/*v%*. The dependent variables were vesicle size (VS) (Y1), ZP (Y2), and EE% (Y3). Table 7 shows the independent (low and high level) and dependent variables. Table 1 shows the composition of QTF-loaded GLSMs.

### 3.3. Preparation of QTF Glycerosomal Formulations

GLSMs were prepared by thin film hydration technique [23] using lecithin as a lipid in a concentration of 5 (%*w*/*v*) based on a pre-screening study. Lecithin, cholesterol, and 100 mg QTF were dissolved in 10 mL ethanol in a flask with a round bottom. A rotary evaporator (Buchi Rotavapor R-200, Allschwil, Switzerland) was used to evaporate the organic solvent under reduced pressure at a temperature of 40 °C and 90 rpm. Then, 10 mL phosphate buffer pH (7.4) containing different concentrations of glycerol was used to hydrate the film, followed by sonication for 10 min using an ultrasonicator (Model 3510; Branson Ultrasonics, Danbury, CT, USA).

### 3.4. Evaluation of QTF Glycerosomal Formulations

#### 3.4.1. Measurement of Vesicles Size (VS), Polydispersity Index (PDI), and Zeta Potential (ZP)

Zetasizer Nano ZS instrument (Malvern Instruments, Worcestershire, UK)was used to measure the VS, PDI, and ZP of the prepared QTP-loaded GLSMs at 25 °C after appropriate dilution with distilled water [39,42,48]. The measurements were done in triplicate.

#### 3.4.2. Measurement of Entrapment Efficiency (EE%)

The prepared glycerosomal formulations were centrifuged at 17,000 rpm for 1 h at 4 °C [48] by a cooling centrifuge (SIGMA 3–30 K, Sigma, Steinheim, Germany) to separate glycerosomal vesicles from the un-entrapped QTF. The concentration of QTF in the supernatant was determined after suitable dilutions using a UV spectrophotometer (Shimadzu UV-1800, Kyoto 604-8511, Japan) at the predetermined λ_max_ (254 nm). Validation of the method was done by calculating linearity within a range of concentration of 2.5 to 20 µg/mL (R^2^ of 0.9994).

The EE% was calculated applying the equation [29,41,42].

(4) $$\mathrm{EE}\%=\frac{TD-FD}{TD}\times 100$$

where EE% is the entrapment efficiency, *FD* and *TD* are the amounts of the free and total drugs, respectively.

The obtained nanoparticles in the bottom of the centrifuge tube were washed with phosphate buffer pH 7.4 and recentrifuged to remove the unentrapped drug. The washing of nanoparticles was repeated in triplicate to ensure the complete removal of the unentrapped drug. The purified nanoparticles were kept for further characterization.

### 3.5. Statistical Analysis, Optimization, and Validation

Factorial analysis of variance (ANOVA) was used to analyze the studied responses using Design Expert^®^ software. A desirability function was used to select the optimum formula with the smallest VS and the highest ZP and EE%. For checking the validity of the used statistical models, The optimum formula was prepared and evaluated for VS, ZP, and EE% and percentage relative errors were calculated between the obtained results and the predicted values using the following equation [34].

(5) $$\%\;\mathrm{Relative}\;\mathrm{error}=\frac{\mathrm{predicted}\;\mathrm{value}\;-\;\mathrm{experimental}\;\mathrm{value})}{\mathrm{predicted}\;\mathrm{value}}\times 100$$

### 3.6. Evaluation of the Optimum QTF Formula

#### 3.6.1. Differential Scanning Calorimetry (DSC)

DSC analysis was accomplished for pure QTF, a physical mixture of lecithin, cholesterol, and QTF, and the optimum formula using a differential scanning calorimeter (DSC N-650; Scinco, Liguria, Italy). Samples of about 5 mg were placed in the aluminum pan of the apparatus and subjected to heat at a rate of 10 °C/minute until 200 °C underflow of inert nitrogen.

#### 3.6.2. X-ray Diffraction Study (XRD)

X-ray diffraction measurements of pure QTF and the optimum formula were performed using an Ultima IV Diffractometer (Rigaku Inc. Tokyo, Japan at College of Pharmacy, King Saud University, Riyadh, KSA). The XRD spectra were scanned in the range of 0–60° (2θ) at a rate of 10°/min speed.

#### 3.6.3. Transmission Electron Microscopy (TEM)

A transmission electron microscope (TEM; JEOL JEM-1010, Tokyo, Japan) was used to visualize the morphology of the optimum formula as well as the dimensions of GLSMs. After diluting the samples suitably, they were put on a carbon-coated copper grid. Then, 2% *w*/*v* phosphotungstic acid was used to coat the samples. They were then kept in the air for 5 min to be dried. Then, a TEM operated under an acceleration voltage of 80 kV [49] and ×80,000 power of magnification was used to image the samples at room temperature. The measurement was repeated six times to calculate the average of GLSMs dimensions.

#### 3.6.4. In-Vitro Release

The release of QTF from the optimum glycerosomal formula compared to drug suspension was studied by placing an amount of each formula equivalent to 5 mg QTF in the dialysis bags. Then it was suspended in a 250 mL dissolution medium (phosphate buffer pH (7.4)) [50] in the dissolution apparatus (Pharm Test, Hainburg, Germany) at a temperature of 37 °C with stirring at 100 rpm. The amount of QTF was quantified at different time points by withdrawing 5 mL from the dissolution media at 1, 2, 3, 4, 5, and 6 h and instantly replaced with an equal amount of fresh media. Then, the concentration of QTF in the collected samples was quantified using a UV spectrophotometer at 254 nm. The measurements were done three times, and the percent of QTP released at different time points was determined as follows [51]:

(6) $$\mathrm{Qn}=\frac{Cn\times Vr+\sum_{i=1}^{n-1}Ci\times Vs}{initial\;drug\;content}$$

where Qn: Percent of QTF released cumulatively

*Cn*: Concentration of QTP in the dissolution medium at the *n*th sample

*Vr*: Volume of dissolution medium

*Vs*: Volume of sample

$\sum_{i=1}^{n-1}Ci$: The summation of the concentrations measured previously

The release profile of the optimum QTF-loaded GLSMs in comparison to drug suspension was made by making a plot between the percentage of QTF released (Q_n_) at different time points vs. corresponding time.

#### 3.6.5. Effect of Aging

The stability of the optimum QTF-loaded GLSMs was assessed as a function of time regarding VS, ZP, and EE% after keeping the formulation in an air-tight vial, kept away from light at 4 °C for one month [52].

### 3.7. In-Vivo Bioavailability of the Optimized QTF Loaded GLSMs

#### 3.7.1. Study Design

The study was done on male Wistar albino rats weighing 140 ± 20 g. They were kept in a temperature-controlled room (22 ± 2 °C) in cages of polypropylene. Standardized pellet feed and clean drinking water were supplied to them. The study was approved by the Institutional Animal Ethical Committee (IAEC) number 202010001 of CPCSEA (Committee for Control and Supervision of Experiments on Animals), Prince Sattam Bin Abdulaziz University. A total of 96 rats were used in the study. They were divided into two groups. The first group was for QTF aqueous suspension and the second group was for the optimum QTF-loaded GLSMs. All animals were fasted for 18 h before receiving any doses. Dosing animals orally is done by the method described by Kuentz [53]. For each group, six animals were kept as control and the rest of the animals received an oral dose equivalent to 20 mg/kg body weight of QTF suspension and optimum QTF-loaded GLSMs, respectively [54,55]. At different time intervals 1, 2, 3, 4, 6, 12, and 24 h following administration of both QTF suspension and optimum QTF loaded GLSMs, six animals were sacrificed from each group by being cervically decapitated, followed by the collection of blood in commercially available anti-coagulant treated tubes for plasma separation. The tubes containing spray-dried Heparin/EDTA anticoagulants are used to separate plasma from the blood. The tube was centrifuged at 2000× *g* for 10 min. Ref. [56] a refrigerated centrifuge was used to separate cells from the plasma by centrifugation for 10 min at 1000–2000× *g*. After that, plasma was immediately conveyed using a Pasteur pipette into polypropylene. While handling the samples, they should be kept at 2–8 °C. Plasma was divided into 0.5 mL aliquots and stored at –20 °C or lower for further use [56]. While the brain was instantly dissected out and washed with cold saline and known amounts of tissues were homogenized at 5000 rpm with appropriate ice-cold buffer in a Teflon homogenizer for 10 min. The plasma and homogenized brain samples were subjected to HPLC evaluation for absorbed quetiapine.

#### 3.7.2. HPLC Assay of QTF in Plasma and Brain

To prepare the serum samples for HPLC analysis, 10 µg/mL of lamotrigine (internal standard) and 0.1 mL of NaOH (0.1 M) were added to 100 µL of serum, and the Valcon tubes were shaken for 1 min as the first step. For the second step, 5 mL of diethyl ether was added and vortexed for 5 min and mixed for 5 min, and the mixtures were then centrifuged at 3000 rpm for 6 min at room temperature. For the third step, 4 mL was carefully suctioned from the upper layer of ether; then, the remaining mixture was extracted once again using 5 mL of diethyl ether. For the fourth step, 4 mL was carefully suctioned from the upper layer of ether and then added together with the previous extract; the evaporation step was done at room temperature. Finally, the reconstitution of the residue with 100 µL of methanol was done then reconstituted samples were injected into the HPLC system [10].

For brain samples, about 1 mL of phosphate buffer (pH 3) was added to the brain homogenate, followed by vortexing. After that, 1 mL of 60% chloroform and 40% of methanol mixture was added to homogenate and mixed for 1 min, then centrifugation at 5000 rpm for 10 min at 4 °C. After that, the organic layer was separated into a tube, then the drug was extracted once again, and the extract was added to the previous one, followed by evaporation under vacuum. Finally, the residue was resuspended in 2 mL HPLC grade of 80% acetonitrile and 20% methanol mixture and then reconstituted samples were injected into the HPLC system [57].

For quantitative estimation of Quetiapine Fumarate in serum and brain samples, a Shimadzu HPLC system (SHIMADZU 1200 series HPLC system (Kyoto, Japan) equipped with a quaternary pump, online degasser, an autosampler (SHIMADZU1200, Kyoto, Japan) (model 20A), and separation in the final method was achieved on a Thermosil^®^ C-18 column (250 mm × 4.6 mm i.d., 5 µm particle size) column (Thermo, USA). The operating temperature of the oven column was fixed at 30 °C. The system was equipped with SPD-20A/20AV UV-Vis detectors set at 254 nm. Isocratic elution was utilized with a mobile phase of 0.02 M of phosphate buffer (pH 5.5) mixed with acetonitrile in a ratio of 35:65. Finally, the 0.45 µm membrane filters were used to filter the mobile phase, then degassed by sonication for 15 min, prior to its use. The injection volume was 20 µL, and the flow rate was 1 mL/min with a total run time of 15 min. The liquid chromatography instrument was interfaced with a computer running LabSolutions software using Microsoft Windows 7. The concentrations of QTF in rat serum and brain samples were compared against a standard of QTF in the mobile phase [58].

#### 3.7.3. Pharmacokinetic Analysis

A plot of the mean QTF plasma concentrations and brain concentrations was made against time. Both plasma and brain Pharmacokinetic parameters were calculated using WinNonlin software (version 1.5, Scientific Consulting, Inc., Rockville, MD, USA). Pharmacokinetic parameters include the peak plasma and brain concentrations (C_max_) in addition to the time to reach these peaks (T_max_). Additionally, the area under the curve till the last time (AUC_0–24_) and till infinity (AUC_0–∞_) were determined using the trapezoidal rule. Moreover, the mean residence time (MRT) and the elimination half-life (T_1/2_) were calculated. Results were expressed as mean values ± standard deviations. Then, ANOVA was used to analyze the obtained pharmacokinetic parameters to test the significant differences between both QTF suspension and optimum QTF-loaded GLSMs.

## 4. Conclusions

Glycerosomes (GLSMs) are a new generation of liposomes containing a high concentration of glycerol (10–30 *w*/*v*%). GLSMs have advantages over liposomes in being more stable and having greater fluidity than liposomes due to the presence of glycerol in high percent. This increased fluidity makes GLSMs more penetrable into the brain tissue than liposomes. GLSMs were prepared and subjected to an optimization process using a Face centered rotatable design on selecting the formula having the smallest vesicle size, the largest zeta potential, and entrapment efficiency. The optimum formula, which was composed of 29.645 *w*/*v*% glycerol, 0.8% cholesterol, and 5% lecithin, showed a vesicle size of 290.4 nm, a zeta potential of −34.58, and entrapment efficiency of 80.85%. It was also revealed that spherical vesicles by TEM with no aggregates indicated high stable systems that are confirmed by the stability study. Additionally, the optimum formula showed enhanced drug release when compared to a drug suspension. Moreover, it was subjected to a pharmacokinetic study where it showed enhanced brain and plasma bioavailability of QTF when compared to the drug suspension. Therefore, it could be concluded that QTF-loaded GLSMs are a promising new nanocarrier for the oral delivery of QTF.

## Figures and Tables

**Table 1:** Composition of Different Coded formulations with their responses in Central Composite Design for optimization of QTF loaded GLSMs.

Formula Code	Independent Variables			Dependent Variables		
	Glycerol conc (*w*/*v*%) (X1)	Cholesterol conc (*w*/*v*%) (X2)	VS (nm) (Y1)	PDI	ZP (mV) (Y2)	EE% (Y3)
G1	5.86	0.5	110.23 ± 6.48	0.248 ± 0.067	−20.3 ± 0.92	28.31 ± 1.74
G2	10	0.2	130.25 ± 7.47	0.268 ± 0.142	−21.8 ± 1.73	43.21 ± 3.62
G3	10	0.8	161.55 ± 10.72	0.174 ± 0.054	−27.35 ± 2.61	54.42 ± 2.18
G4	20	0.08	115.42 ± 5.43	0.125 ± 0.021	−19.1 ± 1.23	32.3 ± 1.26
G5	20	0.5	238.02 ± 4.73	0.402 ± 0.023	−30.4 ± 2.42	66.2 ± 2.81
G6	20	0.92	283.56 ± 11.23	0.281 ± 0.126	−34.4 ± 1.89	73.2 ± 1.34
G7	30	0.2	232.30 ± 7.82	0.265 ± 0.134	−31.4 ± 2.26	65.79 ± 2.64
G8	30	0.8	321.51 ± 4.73	0.345 ± 0.084	−37.7 ± 1.82	78.08 ± 3.21
G9	34.14	0.5	228.42 ± 6.29	0.352 ± 0.078	−29.1 ± 2.35	64.3 ± 4.32

VS: vesicle size, ZP: zeta potential, PDI: polydispersity index, EE%: entrapment efficiency percent, Data represented as mean  ±  SD (*n*  =  3).

**Table 2:** Output data of Central Composite Design of QTF loaded GLSMs.

Dependent Variables	R^2^	Adjusted R^2^	Predicted R^2^	Adequate Precision
Y1: VS (nm)	0.8393	0.7858	0.6387	9.6576
Y2: ZP (mV)	0.8245	0.7660	0.5859	9.1957
Y3: EE %	0.7880	0.7174	0.5272	8.1482

VS: vesicle size, ZP: zeta potential, EE%: entrapment efficiency percent.

**Table 3:** ANOVA for Central Composite Design of QTF loaded GLSMs.

Dependent Variable	Source	SS	Df	Mean Square	F Value	*p*-Value
Y1	Model	39,069.40	2	19,534.70	15.67	0.0041
	X1	23,020.78	1	23,020.78	18.47	0.0051
	X2	16,048.63	1	16,048.63	12.87	0.0115
Y2	Model	271.36	2	135.68	14.10	0.0054
	X1	131.18	1	131.18	13.63	0.0102
	X2	140.18	1	140.18	14.56	0.0088
Y3	Model	2006.51	2	1003.26	11.15	0.0095
	X1	1179.46	1	1179.46	13.11	0.0111
	X2	827.05	1	827.05	9.19	0.0230

Y1: VS (nm), Y2: ZP (mV), Y3: EE%, X1: Glycerol concentration (*w*/*v*%), X2: Cholesterol concentration (*w*/*v*%), SS: sum of squares, df: degree of freedom.

**Table 4:** Validation of the optimum formula.

	VS (nm)	ZP (mV)	EE%
Predicted value	298.88	−35.997	78.08
Experimental value	290.4	−34.58	80.85
% Relative error	2.84	3.94	3.55

**Table 5:** The effect of storage at 4 °C for one month on VS, ZP, and EE% of the optimized formula.

Responses	Fresh	After 7 Days	After 30 Days
VS (nm)	290.41 ± 10.43	292.93 ± 13.43	300.34 ± 12.38
ZP (mV)	−34.58 ± 2.13	−34.24 ± 1.88	−33.67 ± 1.65
EE%	81.23 ± 2.43	80.85 ± 3.98	79.46 ± 3.01

**Table 6:** Pharmacokinetic Parameters of QTF in the brain after oral administration of QTF suspension and QTF Loaded GLSMs.

Pharmacokinetic Parameters	Brain Data		Plasma Data	
	QTF Suspension	QTF Loaded GLSMs	QTF Suspension	QTF Loaded GLSMs
t_1/2_ (h)	13.009 ± 2.59	12.835 ± 5.88	31.291 ± 3.783	47.859 ± 17.880
T_max_ (h)	4.000 ± 0.00	4.666 ± 1.15	2.666 ± 0.577	3.333 ± 0.577
C_max_ (µg/mL)	33.393 ± 4.33	49.806 ± 11.69	8.933 ± 2.656	14.953 ± 8.304
AUC_0–24_ (µg.h/mL)	318.126 ± 13.82	489.753 ± 41.78	131.998 ± 12.020	178.406 ± 6.108
AUC_0–∞_ (µg.h/mL)	496.187 ± 39.28	759.934 ±167.91	341.538 ± 19.888	614.155 ± 169.148
MRT (h)	21.983 ± 4.19	21.949 ± 8.70	46.772 ± 6.694	69.418 ± 25.772
% Bioavailability Enhancement	153.15		179.82	

C_max_: maximum plasma concentration, T_max_: time to reach maximum plasma concentration, AUC: the area under the curve; MRT: mean residence time. Data represented as mean  ±  SD (*n*  =  3).

**Table 7:** Central Composite Design for optimization of QTF loaded GLSMs.

Independent Variables	Levels	
	Low	High
Glycerol concentration *w*/*v*% (X1)	10	30
Cholesterol concentration *w*/*v*% (X2)	0.2	0.8
Dependent values (Responses)	Desirability	
Vesicle size (Y1)	Minimize	
Zeta potential (Y2)	Maximize	
Entrapment efficiency (Y3)	Maximize	

## Data Availability

The data is contained in the manuscript.
